# Hemodynamic management of a patient with a huge right atrium myxoma during thoracic vertebral surgery: A case report

**DOI:** 10.1097/MD.0000000000012543

**Published:** 2018-09-28

**Authors:** Haitao Jia, Yanhong Xing, Shuangyin Zhang, Yingbin Wang

**Affiliations:** Department of Anesthesiology, Lanzhou University Second Hospital, Lanzhou, China.

**Keywords:** hemodynamic change, myxoma, prone position, right atrium

## Abstract

**Rationale:**

Myxoma is the most common primary benign cardiac tumor, which could lead to some fatal complications because of its strategic position.

**Patient concerns:**

The patient was admitted to our hospital due to sudden onset of palpitation, chest tightness, mild fever, night sweats, accompanied with bilateral lower extremities adynamia, and paralysis for 5 days, but no obvious syncope and edema.

**Diagnoses:**

Transthoracic echocardiography showed a giant mobile myxoma (72 × 58 mm) in the right atrium (RA). Magnetic resonance imaging revealed an erosive space-occupying lesion located between the first and third thoracic vertebrae.

**Interventions:**

Thoracic vertebral lesions were resected immediately to rescue the incomplete paraplegia. After the patient was placed in the prone position, significant hemodynamics changes were observed due to the displacement of the huge RA myxoma.

**Outcomes:**

Stable hemodynamics was maintained during the operation through control of fluid infusion combined with vasoactive drugs.

**Lessons:**

Change in body position may lead to obstruction of intracardiac blood flow in patients with giant myxoma. This clinical manifestation is rarely reported.

## 1 Introduction

Myxoma usually originates from the endocardium, especially in the fossa ovalis. Myxoma is the most common primary cardiac tumor.^[1,2]^ Although myxoma is a benign tumor, it can also lead to some fatal complications because of its strategic position. Clinical features typically include intracardiac blood flow obstruction and embolism, which are related to the size, position, and activity of the tumor.^[3]^ In this report, the patient had incomplete paraplegia which rapidly progressed in 5 days because of thoracic vertebral tumor. He was subsequently admitted to our hospital. Moreover, preoperative echocardiography revealed a huge mobile myxoma located in the right atrium (RA), obstructing the intracardiac flow in prone position during thoracic vertebra surgery. Herein, we present our case findings in detail.

## 2 Case presentation

**Figure 1:**
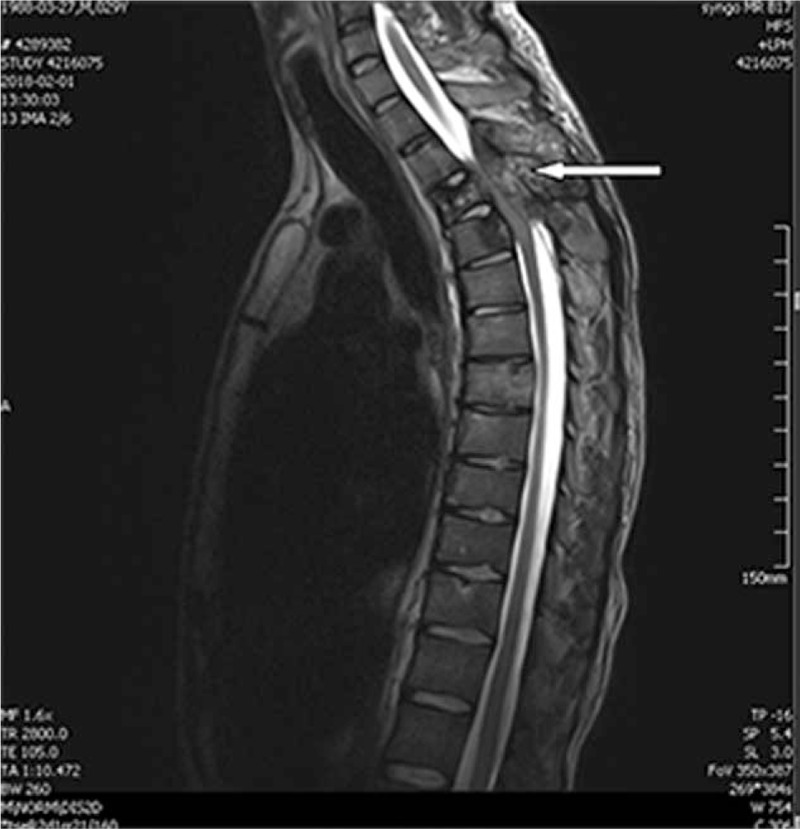
Magnetic resonance images demonstrate first, second, third thoracic vertebrae, and appendage bone destruction and forming a lump. Spinal canal narrowing and thinning (arrow).

**Figure 2:**
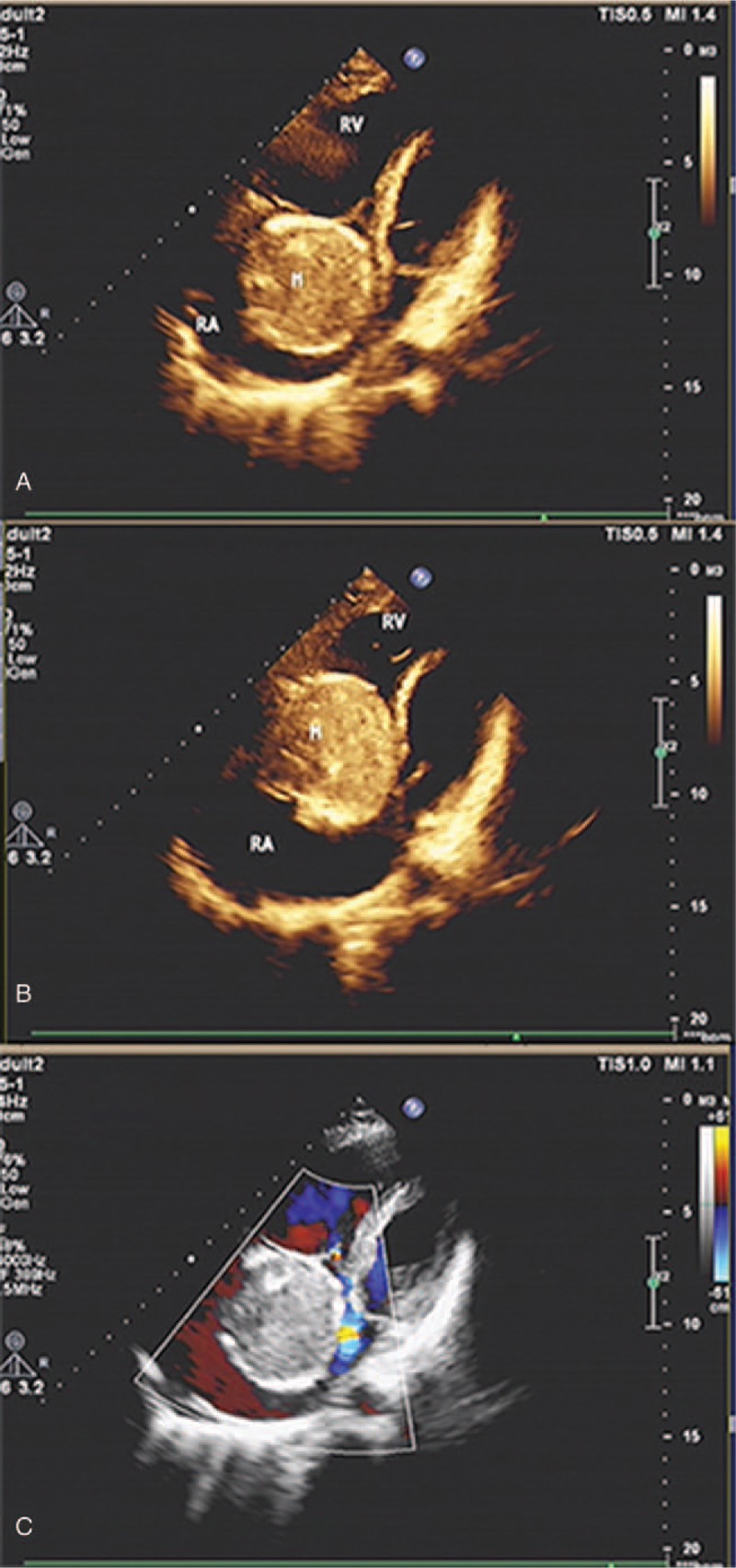
Transthoracic echocardiography demonstrating a huge, regular mass (72 × 58 mm) located at the right atrium in the systole (A) and prolapse to the right ventricle in diastole (B). The tumor causes in mild stenosis and moderate regurgitation of the tricuspid valve (C). M = mass, RA = right atrium, RV = right ventricle.

A 29-year-old man (height = 180 cm, weight = 60 kg) was admitted to our hospital due to sudden onset of palpitation, chest tightness, mild fever, and night sweats, accompanied with bilateral double lower extremities adynamia and paralysis for 5 days, but no obvious syncope and edema. Examination on admission revealed a normal heart rate of 96 beats/minute (bpm) and blood pressure of 120/80 mmHg. On cardiac auscultation, 3/6 grade systolic murmur (Levine Scale) was heard between the third and fourth ribs at the left margin of sternum. The sensory below the sternum was dysfunctional. Muscle strength on both legs was at 1/6 levels (Lovett Scale), and tendon reflex diminished. Electrocardiography showed a sinus rhythm with pulmonary P-wave. An X-ray image of the chest showed discrete and scattered miliary nodules over both lungs, and cardiac silhouette was enlarged. Magnetic resonance imaging (MRI) (Fig. 1) showed an erosive space-occupying lesion located between the first and third thoracic vertebrae, which resulted in stenosis of the spinal canal and thinning of the spinal cord. Transthoracic echocardiography (TTE) (Fig. 2) showed a huge mobile mass (72 × 58 mm) in the RA and myxoma was considered. The left ventricular ejection fraction was 60%. The remaining physical examination findings were unremarkable, and laboratory tests were normal, except for the accelerating erythrocyte sedimentation rate. There was no family history of heart disease, including tumors and other cardiovascular problems. The preliminary diagnosis was thoracic vertebra tumor and cardiac myxoma. To treat the incomplete paraplegia caused by thoracic vertebra tumor erosion, the operation including resection of thoracic vertebral lesions, decompression, and internal fixation with nail-rod must be carried out immediately. A multiple disciplinary team consisting of cardiologist, orthopedist, sonologist, and anesthesiologists, was created. Fatal complications of cardiac myxoma, such as intracardiac obstruction and pulmonary embolism, were assessed. Informed consent of critical illness was signed by the patient's legal representative.

The patient did not receive premedication before anesthesia. On arrival in the operating room, peripheral venous access was established on the dorsum of the hand, and continuous electrocardiogram and pulse oximetry monitoring were instituted. Invasive arterial blood pressure (IBP) was measured in the left radial artery, and the central venous pressure (CVP) was measured in the right femoral vein. Anesthesia induction was carried out with intravenous injection of midazolam 4 mg, penehyclidine hydrochloride 1 mg, etomidate 16 mg, and sufentanil 50 μg. Cisatracurium 12 mg was used to facilitate tracheal intubation. Maintenance of compound anesthesia consisted of 2% sevoflurane in an air–oxygen mixture, and remifentanil was continuously injected intravenously at 0.2 μg/kg/min. Intermittent bolus dose of 5 mg cisatracurium was administered for muscle relaxation. After anesthesia induction, hemodynamic parameters were stable at the supine position: heart rate of 80 bpm, IBP of 110/65 mmHg, and CVP of 8 mmHg. However, after the patient was turned to the prone position on the level of the standard operating table (lying on a pair of bolsters, unpressurized abdomen), the patient's heart rate increased gradually to 130 bpm, IBP dropped to 70/45 mmHg, and CVP increased to 22 mmHg. Moreover, the jugular vein was filled observably. Emergency treatment was carried out immediately against the symptoms of right heart failure. Fluid infusion was restricted and vasoactive drugs were used to maintain appropriate arterial blood pressure. Dopamine at 5 to 10 μg/kg/min and norepinephrine at 0.03 to 0.05 μg/kg/min were continuously injected intravenously, keeping the mean arterial pressure above 70 mmHg during the operation. During the 3 hours of surgery, blood volume supplementation totally consisted of sodium chloride 500 mL, hydroxyethyl starch 500 mL, fresh-frozen plasma 600 mL, and suspended red blood cells 2 U. Total blood volume loss was 600 mL, and urine output was 500 mL. After the operation, anesthetic drugs were stopped, and the patient was returned to the supine position. At this moment, his hemodynamic status was stabilized gradually with a heart rate of 84 bpm, IBP of 130/88 mmHg, and CVP of 10 mmHg. Dopamine and norepinephrine intravenous titration was suspended. When the patient recovered spontaneous respiration and responded to verbal commands, tracheal extubation was performed. After ventilated with 100% oxygen via a facemask in 5 min, the patient was transferred to the post-anesthesia care unit. Evaluation of spinal function was performed on the second postoperative. The muscle strength of the left leg was 4/6 levels, and the right leg was 3/6 levels; meanwhile, the superficial sensibility was considered dysesthesia. Furthermore, hemodynamic parameters were stable at the ward.

## 3 Discussion

Myxoma is the most common cardiac tumor, which accounts for half of the primary cardiac neoplasms, and 75% of them originate from the left atrium and 20% occur in the RA, but origin in the ventricle is rare.^[4]^ Clinical reports of cardiac myxoma are more prevalent in middle-aged women, and only a few patients have a family history.^[5,6]^ Most of the myxomas are single lesion, and a few cases present 2 or more tumors in the same or different cardiac cavity.^[7]^ Although myxoma is a benign neoplasm in histology, it can also lead to some critical complications, such as intracardiac blood flow obstruction, pulmonary hypertension, hypoxemia, congestive heart failure, embolism complication, infection, malignant change, and so on.^[3,4,8,9]^ Left atrial myxoma can lead to systemic circulation embolism, which most commonly occurs in cerebral vessels.^[5,10,11]^ Right atrial myxoma could lead to pulmonary embolism, pulmonary hypertension, and Budd–Chiari syndrome.^[12,13]^ A large mobile myxoma could suddenly block the atrioventricular or pulmonary valves, causing thoracodynia, syncope, convulsion, and even sudden death. The differential diagnosis includes constrictive pericarditis, rheumatic tricuspid valve stenosis, bacterial endocarditis, and myocardiopathy.^[3]^ However, a report revealed a huge myxoma in the RA without any clinical symptoms.^[14]^

The clinical manifestation of myxoma is related to the size, shape, location, and activity of the tumor. The displacement of myxoma may result in changes of intracardiac hemodynamics, and clinical symptoms are either aggravated or reduced accordingly. Some cases without significant systemic symptoms and discovered incidentally by echocardiographic examination could cause fever or increase erythrocyte sedimentation because the myxoma releases inflammatory cytokines.^[15]^ In the present case, the huge myxoma of the RA was about 72 × 58 mm in size, which is very rare. The patient's hemodynamic status was stable in the supine position after anesthesia induction, but it significantly changed when he was positioned prone. In addition, the CVP was increased significantly, and the jugular vein was filled remarkably.

In the present case, we have excluded the effect of anesthesia and mechanical ventilation and rejected the possibility of increasing intraperitoneal pressure that affects CVP in prone position.^[16]^ We could not evaluate the heart function by transthoracic or transesophageal echocardiography in real time because of the restriction of the prone position, but we could judge that there was a sudden and critical right heart failure based on the clinical manifestations. The significant changes in hemodynamics were definitely due to the displacement of the huge RA myxoma. The prone position aggravated the incomplete obstruction in the right heart chamber, which was reduced when the patient was repositioned to supine. Thereafter, the hemodynamics gradually stabilized and vasoactive drugs were not needed continuously. Therefore, under fluid infusion control combined with vasoactive drugs, we maintained stable vital signs during the operation and ensured successful completion of the operation, which saved valuable time for recovery of spinal cord function. All of this confirmed our previous speculation and improved the experience for management of anesthesia in patients with myxoma in future.

Echocardiography is an important method for the diagnosis of myxoma and assessment of cardiac function, with high specificity.^[3,17]^ Because of the restriction of the body position, we failed to assess the heart function in real time by transthoracic or transesophageal echocardiography during the operation, which is the limitation of this case report. Once the cardiac myxoma is diagnosed, it should be treated immediately. The success rate of thoracotomy resection with total cardiopulmonary bypass is almost 80%, and the recurrence rate is 3% to 5%.^[18,19]^ In addition, follow-up every 6 months after the operation is necessary with further investigation by echocardiography.^[17,20]^ Unfortunately, because the vertebral tumor was diagnosed as malignant cancer and had multiple lung metastases, the patient refused thoracotomy for cardiac myxoma and was discharged automatically after a week.

## 4 Conclusion

The clinical feature of the myxoma typically includes intracardiac blood flow obstruction and embolism, which are related to the size, position, and activity of the tumor. In the present case, the hemodynamics was stable in the supine position but significantly changed when the patient was turned to the prone position. This shows that a change in body position could aggravate the intracardiac obstruction, and the clinical symptom can be either aggravated or reduced accordingly. This clinical manifestation of myxoma is rarely reported.

## Acknowledgments

We would like to acknowledge the patient and his family for allowing us to present the disease course in this report. Written informed consent was obtained from the patient for publication of this case report and accompanying images. Moreover, we would like to thank Editage (www.editage.com) for English language editing.

## Author contributions

Conceptualization: Haitao Jia.

Investigation: Yanhong Xing.

Supervision: Shuangyin Zhang.

Writing – original draft: Haitao Jia.

Writing – review & editing: Yingbin Wang.
